# Intake of Baked Cod Fillet Resulted in Lower Serum Cholesterol and Higher Long Chain *n*-3 PUFA Concentrations in Serum and Tissues in Hypercholesterolemic Obese Zucker fa/fa Rats

**DOI:** 10.3390/nu10070840

**Published:** 2018-06-28

**Authors:** Linn A. Vikøren, Aslaug Drotningsvik, Marthe T. Bergseth, Svein A. Mjøs, Maren H. Austgulen, Gunnar Mellgren, Oddrun A. Gudbrandsen

**Affiliations:** 1 Dietary Protein Research Group, Department of Clinical Medicine, University of Bergen, 5021 Bergen, Norway; linn.anja.slake.vikoren@helse-bergen.no (L.A.V.); aslaug.drotningsvik@uib.no (A.D.); marberg87@gmail.com (M.T.B.); 2 Department of Clinical Science, University of Bergen, 5021 Bergen, Norway; maren_austgulen@hotmail.com; 3 Department of Chemistry, University of Bergen, P.O. Box 7803, 5020 Bergen, Norway; svein.mjos@uib.no; 4 Nofima BioLab, P.O. Box 1425 Oasen, 5828 Bergen, Norway; 5 Department of Clinical Science, KG Jebsen Center for Diabetes Research, University of Bergen, Haukeland University Hospital, 5021 Bergen, Norway; gunnar.mellgren@uib.no; 6 Hormone Laboratory, Haukeland University Hospital, 5021 Bergen, Norway

**Keywords:** cholesterol, cod, fish fillet, *n*-3 PUFA, obese Zucker rat

## Abstract

Increasing evidence indicates that lean fish consumption may benefit cardiovascular health. High cholesterol and low *n*-3 PUFA concentrations in serum are associated with an increased risk of coronary heart disease; therefore, it is of interest to investigate effects of cod intake on cholesterol and *n*-3 PUFAs in serum and tissues. Hypercholesterolemic obese Zucker fa/fa rats were fed diets containing 25% protein from baked cod fillet and 75% protein from casein (Baked Cod Diet), or casein as the sole protein source (Control Diet) for four weeks. Consuming Baked Cod Diet resulted in lower serum cholesterol and lower hepatic mRNA concentrations of HMG-CoA reductase and sterol O-acyltransferase-2 without affecting serum bile acid concentration, faecal excretion of cholesterol and bile acid, and hepatic concentrations of bile acids, cholesterol and cholesterol 7 alpha-hydroxylase mRNA when compared to Control Diet. Rats fed Baked Cod Diet had higher concentrations of *n*-3 PUFAs in serum, liver, skeletal muscle and adipose tissue. To conclude, baked cod fillet intake resulted in lower serum cholesterol, which was probably caused by lower endogenous cholesterol synthesis, and higher *n*-3 PUFA in serum and tissues in obese Zucker fa/fa rats. These findings support the evidence that lean fish consumption might benefit cardiovascular health.

## 1. Introduction

More than 1.9 billion adults were overweight in 2014, and of these 600 million were obese according to The World Health Organization. The prevalence of overweight and obesity has more than doubled since 1980, and has become a worldwide problem affecting both high-, middle-, and low-income countries [1]. The increased prevalence of overweight and obesity is of major concern as they are strongly associated with high cholesterol [2] and predispose for diseases such as cardiovascular diseases which today are considered a large contributor to morbidity and mortality globally [1,3]. High cholesterol is a strong predictor of vascular mortality [4], and lowering serum cholesterol concentration is an important strategy for reducing the risk of cardiovascular disease [5].

Intake of fish and fish oil have been associated with lower circulating cholesterol concentrations in several but not all studies [6,7,8,9,10,11,12,13,14,15,16,17,18,19], and fish consumption is associated with reduced risk of coronary heart disease and heart failure [20,21,22,23]. The health benefits of fish consumption have traditionally been attributed mainly to the effect of n-3 polyunsaturated fatty acids (PUFA), however there is controversy as to the cholesterol-regulating effects of fish oil and the marine n-3 fatty acids in humans [6,7,8,9,10,11]. Lean fish contains very little long chain *n*-3 PUFAs, but still cod fillet intake seems to have beneficial effects on circulating cholesterol in humans [15,24,25], suggesting that there are other components in fish such as fish proteins that may be advantageous to human health and affect risk factors leading to cardiovascular disease beyond the *n*-3 PUFAs. In line with this, we have recently found that cod protein supplementation reduced LDL cholesterol in overweight and obese healthy adults [26], and this is supported by results from animal studies showing that cod proteins have beneficial effects on cholesterol concentrations [27,28,29].

The obese Zucker fa/fa rat presents changes often seen in human obesity, and is a representative rat model for studies of metabolic complications and possible treatments of obesity [30]. Visible obesity is present already 3-5 weeks after birth, and in addition the obese Zucker rats develop a range of endocrine abnormalities resembling human metabolic syndrome such as dyslipidaemia, insulin resistance, mild glucose intolerance and hyperinsulinemia [30]. Obese Zucker rats have serum cholesterol concentration approximately twice that of lean Zucker rats already when they are 11 weeks old [31], making this an interesting animal model for studies on the effects of diets on cholesterol metabolism.

Higher serum and tissue concentrations of long-chain *n*-3 PUFAs are associated with reduced risk of coronary heart disease events [32]. Consumption of cod fillet has been reported to increase *n*-3/*n*-6 PUFA ratio in plasma phospholipids in humans [33], and we have recently shown that obese Zucker fa/fa rats fed cod protein also had higher ratio of *n*-3/*n*-6 PUFAs in serum, liver and adipose tissue [34]. 

Most studies on the effects of fish intake in animals are conducted with fish oils, pure *n*-3 PUFAs or fish proteins, and studies on how fish fillet as part of a normal diet affects cholesterol and fatty acids in rats are warranted and relevant for human fish consumption. We have recently shown that diets containing raw and baked salmon fillet had very similar effects on lipid metabolism in obese Zucker fa/fa rats, with marginally more marked effects of the diet containing baked salmon [35]. Salmon is frequently prepared by heat treatment and is also a popular ingredient in its raw form in sushi, whereas cod is more commonly consumed after heat treatment. Therefore, the objective of the present study was to investigate the effects of feeding obese Zucker fa/fa rats an AIN-93G diet containing baked cod fillet in an amount corresponding to 25% of total protein intake on regulation of cholesterol and *n*-3 PUFAs. We hypothesized that cod fillet intake would lower serum cholesterol and increase contents of long chain *n*-3 PUFAs in serum and tissues in this obese rat model with hypercholesterolemia. 

## 2. Materials and Methods

### 2.1. Animals and Diets

Twenty male Zucker fa/fa rats (HsdHlr:ZUCKER-Leprfa, from Harlan Laboratories, Indianapolis, IN, USA) were randomly assigned to two experimental groups of ten rats each with comparable mean body weight. Rats were housed in pairs in Makrolon IV cages in a room maintained at a 12 h light–dark cycle (light from 7 a.m. to 7 p.m.) with constant temperature of 20–23 °C and relative humidity of 65 ± 15%. The rats were acclimatized under these conditions before the start of the experiment.

The intervention period started when the rats were 8–9 weeks old and weighing 355 ± 10 g (mean ± SD). The rats were fed experimental diets based on AIN-93G [36] (Table 1) for four weeks. The Baked Cod Diet contained 5 wt % proteins from cod fillet and 15 wt % proteins from casein. The Control Diet contained 20 wt % proteins from casein. Fillets from Atlantic cod (Gadus morhua) were provided by Lerøy Seafood Group (Hordaland, Norway). Skin free cod fillets were prepared in oven at 180 °C for 20 min, no oil or fat were added when baking the fish. Baked cod fillets were minced, freeze dried and grinded before it was mixed with the other ingredients in the Baked Cod Diet. Diets were immediately frozen after preparation. Casein was purchased from Sigma-Aldrich (Munich, Germany), all other ingredients were purchased from Dyets Inc. (Bethlehem, PA, USA). Rats had free access to tap water and feed (ad libitum). Feed was given as a powder formula and was contained in ceramic bowls that were too heavy for the rats to knock over. Newly thawed feed was provided every day except Sundays (rat were given double doses on Saturdays). Rats always had access to wood chewing sticks and plastic housing. The rats were weighed every seventh day during the intervention period. 

The rats were housed individually in cages with grids for 24 h on day 18 of the intervention period, without fasting in advance, for measurements of feed intake and collection of faeces. 

The rats were euthanized after four weeks intervention while under anaesthesia with Isofluran (Isoba vet, Intervet, Schering-Plough Animal Health, Boxmeer, The Netherlands) mixed with nitrous oxide and oxygen, after a 12 h fast with free access to tap water. The body length (without tail) of the rats was measured with a ruler when rats were anesthetized. Blood was drawn directly from the heart and collected in Vacuette Z Serum Clot Activator Tubes (Greiner Bio-one) for isolation of serum. Liver, thigh muscle, and epididymal, renal and retroperitoneal white adipose tissues (WAT) were dissected out and weighed. Serum and tissues were frozen in liquid nitrogen and stored at −80 °C until analysis. 

Assessors were blinded to diet groups during animal care (feeding, weighing, daily care), euthanasia and analyses of samples.

### 2.2. Ethics

The study protocol was approved by the National Animal Research Authority (Norway) in accordance with the Animal Welfare Act and the Regulation of animal experiments (approval no 2014/6979). All applicable international, national and institutional guidelines for the care and use of animals were followed.

### 2.3. Analyses of Diets

The contents of total amino acids, free amino acids, taurine and total energy in the diets were analysed by Nofima BioLab (Hordaland, Norway). Lipids were extracted from diets using a mixture of chloroform and methanol [37], and fatty acid composition and cholesterol content in the diets were analysed by gas chromatography and spectrometry, respectively, as previously described [35,37,38,39,40,41]. 

### 2.4. Analyses in Serum

Serum concentrations of total cholesterol, LDL cholesterol and HDL cholesterol were analysed by accredited methods at the Laboratory of Clinical Biochemistry at Haukeland University Hospital (Bergen, Norway). Serum total bile acids were analysed on the Cobas c111 system (Roche Diagnostics GmbH, Mannheim, Germany) using the total bile acid assay (Diazyme Laboratories, Inc., California, USA). The EIA-2048 kit (DRG Instruments GmbH, Marburg, Germany) was used for analyses of serum insulin.

### 2.5. Fatty Acids in Serum, Liver, Muscle and Adipose Tissue

Lipids were extracted from liver and skeletal muscle as described by Bligh and Dyer [37] before methylation. Serum and epididymal adipose tissue samples were methylated without prior extraction of lipids. Extracts, serum and adipose tissue were methylated and analysed as described previously [38,39,40,41].

### 2.6. Analyses of Cholesterol in Liver, Muscle and Faeces

Cholesterol was measured in lipid extracts from liver, skeletal muscle and faeces as previously described [37,41].

### 2.7. Bile Acids in Liver and Faeces

Total bile acids (3α-hydroxy bile acids) concentration was measured in freeze-dried faeces [42] using Chromabond C18 ec (3 mL/200 mg, Macherey-Nagel, Düren, Germany) and in liver. Samples were analysed using the enzymatic bile acid assay from Diazyme Laboratories, Inc. on the Cobas c111 system (Roche). Liver protein was measured with the Bradford dye-binding method [43] using Protein Assay Dye Reagent (Bio-Rad Laboratories, Munich, Germany) with bovine serum albumin (Bio-Rad Protein Assay Standard II, Bio-Rad Laboratories, Hercules, CA, USA) as standard. Concentration of total bile acids in liver is calculated relative to protein concentration.

### 2.8. Analyses of mRNA Expression of Genes in Liver

Total RNA was purified from liver using the RNeasy Mini Kit (Qiagen, Hilden, Germany) according to the manufacturer’s protocol. RNA concentration and quality were measured using QIAxpert (Qiagen, Hilden, Germany). High Capacity cDNA Reverse Transcription Kit (Applied Biosystems, Foster City, CA, USA) was used to synthesize cDNA from 150 ng total RNA per sample. cDNA was diluted 1:5 with PCR-grade water before qPCR was performed (in triplicate) using the LightCycler480 rapid thermal cycler system (Roche Diagnostics GmbH, Basel, Switzerland) with the LightCycler 480 SYBR Green I Master (Roche, Basel, Switzerland). The following primer pairs from Sigma were used: HMG-CoA reductase (HMGCR): forward primer 3′GACCTTTCTAGAGCGAGTGCAT’5 and reverse primer 3′CGCTATATTCTCCCTTACTTCATCC’5, Sterol O-acyltransferase 2 (SOAT2); forward primer 3′CCCAGACCTGGTACAATGGA’5 and reverse primer 3′CTGTGCTTGCTCCAGACACT’5, Cholesterol 7 alpha-hydroxylase (CYP7A1); forward primer 3′GCAACCTTTTGGAGCTTATTTC’5 and reverse primer 3′GCACTCTGTAAAGCTCCACTCA’5, LDL receptor (LDLR); forward primer 3′TGCTACTGGCCAAGGACAT’5 and reverse primer 3′CTGGGTGGTCGGTACAGTG’5, Fatty acid desaturase 1 (FADS1); forward primer 3′GAACTCTCTTCTGATTGGAGAGCTA’5 and reverse primer 3′CCGGAATTCATCAGTGAGC’5, Fatty acid desaturase 2 (FADS2); forward primer 3′AATTTCCAGATTGAGCACCAC’5 and reverse primer 3′AGTGGGGCAATCTTGTGC’5, Stearoyl-CoA desaturase-1 (SCD1); forward primer 3′GAAGCGAGCAACCGACAG’5 and reverse primer 3′GGTGGTCGTGTAGGAACTGG’5. Three primer pairs were tested as reference genes: 60S ribosomal protein L32 (RPL32); forward primer 3′GTGGCTGCCATCTGTTTTG’5 and reverse primer 3′TTCTTGGTCCTCTTTTTGACG’5 (Sigma-Aldrich), 60S acidic ribosomal protein P0 (RPLP0); forward primer 3′GATGCCCAGGGAAGACAG’5 and reverse primer 3′GAAGCATTTTGGGTAGTCATCC’5 (Sigma-Aldrich), and 18S ribosomal RNA (18S); forward primer 3′AGTCCCTGCCCTTTGTAC′5 and reverse primer 3′GATCCGAGGGCCTCA’5 (Eurogentec, Seraing, Belgium). Of the tested reference genes, 18S had least variation in readings of samples in triplets and was least affected by the intervention diets. Hepatic mRNA concentrations are presented relative to 18S rRNA and normalised to Control Group.

### 2.9. Outcome Measurements

The primary outcome of this study was differences in circulating cholesterol in obese Zucker fa/fa rats after consumption of Baked Cod Diet. The secondary outcomes were differences in fatty acid composition in serum and tissues, and markers of cholesterol and fatty acid metabolism including gene expression of relevant enzymes.

### 2.10. Sample Size

The present study is considered to be a pilot study, since to our knowledge this is the first study on the effects of baked cod fillet as part of a normal diet for growing rodents (AIN-93G) on cholesterol and fatty acid composition in obese Zucker fa/fa rats. In a similar study using salmon fillet as part of the AIN-93G diet, we used 6 rats in each group [35], but recent experience from clinical trials comparing effects of salmon fillet and cod fillet in normal weight adults [44] and overweight adults [45] suggest that cod fillet may have less potent effects on cholesterol and fatty acids concentrations when compared to salmon fillet. We therefore decided to include 10 rats in the Control Group and 10 rats in the Baked Cod Group.

### 2.11. Statistical Analysis

Statistical analyses were conducted using SPSS Statistics version 25 (SPSS, Inc., IBM company, Armonk, NY, USA). The majority of data were normally distributed according to the Shapiro–Wilk test, and groups were compared using Independent Samples t-test assuming equal variances. The cut-off level for statistical significance was taken at a probability of 0.05. Rats fed a casein-based diet served as controls. Data are presented as mean ± standard deviation. One rat in the Control Group was euthanized due to a wound that did not heal and is not included in the results, therefore n = 9 in the Control Group and n = 10 in the Baked Cod Group. 

## 3. Results

### 3.1. Diets and Anthropometry

The contents of indispensable amino acids, the conditionally essential amino acid arginine [46], the functional amino acid glycine and of fatty acids were comparable between the Baked Cod Diet and the Control Diet (Table 2). The amino sulphonic acid taurine and the long chain *n*-3 PUFAs 20:5n-3, 22:5n-3 and 22:6n-3 were found only in the Baked Cod Diet. Contents of free amino acids in the diets were below level of detection except for methionine which was added in its free form to the diets (data not presented). Ratios of lysine/arginine and methionine/glycine were similar in the Baked Cod Diet and in the Control Diet. Dietary cholesterol content was higher in the Baked Cod Diet when compared to the Control Diet. 

Rats in the Baked Cod Group and the Control Group had a similar bodyweight at baseline, and no significant differences in mean body weight were seen between the groups at 1, 2 and 3 weeks (data not presented) or at endpoint (Table 3). The mean body weight-to-square body length at endpoint, the energy intake, the relative weights of selected WAT depots, liver and thigh muscle as well as dry faecal output per kilo body weight were similar in the two groups. 

### 3.2. Cholesterol and Bile Acids

Circulating concentrations of total, HDL and LDL cholesterols were significantly lower in rats fed the Baked Cod Diet when compared to rats in the Control Group after four weeks intervention (Table 4). The mean serum total bile acid concentration was similar between the groups. 

**Figure 1:**
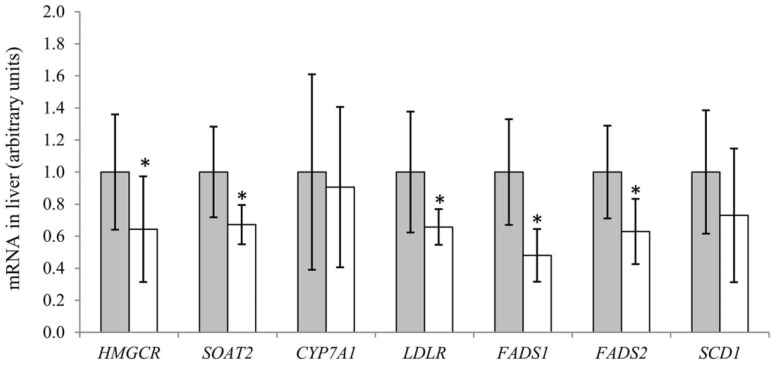
Hepatic mRNA levels in obese Zucker fa/fa rats fed Control Diet (grey bars) or Baked Cod Diet (white bars), presented relative to 18S rRNA and normalised to Control group. Values are mean and standard deviations, n = 9 in Zucker fa/fa Control Group, and n = 10 in Baked Cod Group. * Significantly different from rats fed Control Diet. *p* < 0.05 were considered statistically significant. Groups are compared using Independent Samples t-test assuming equal variances. HMGCR; HMG-CoA reductase, SOAT2; Sterol O-acyltransferase 2, CYP7A1; Cholesterol 7 alpha-hydroxylase, LDLR; Low density lipoprotein receptor, FADS1; Fatty acid desaturase 1, FADS2; Fatty acid desaturase 2, SCD1; Stearoyl-CoA desaturase-1.

The contents of cholesterol and total bile acids in liver were similar between the groups (Table 5), and also no difference was seen between the groups for muscle cholesterol content. The daily faecal excretions of cholesterol and bile acids were also similar between the Baked Cod Group and the Control Group. The hepatic mRNA concentrations of HMGCR, SOAT2 and LDLR were significantly lower in Baked Cod Group when compared to Control Group, whereas no difference was seen for CYP7A1 mRNA in liver between the two groups (Figure 1).

### 3.3. Fatty Acids in Serum, Liver, Skeletal Muscle and White Adipose Tissue

**Figure 2:**
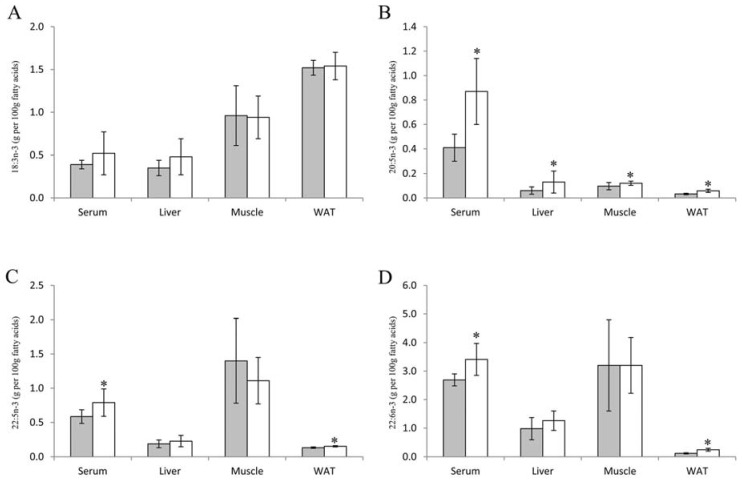
Long chain *n*-3 PUFAs in serum, liver, thigh muscle and epididymal white adipose tissue (WAT) in obese Zucker fa/fa rats fed Control Diet (grey bars) or Baked Cod Diet (white bars). Values are mean and standard deviations, n = 9 in Zucker fa/fa Control Group, and n = 10 in Baked Cod Group. Figure shows contents of 18:3n-3 (**A**), 20:5n-3 (**B**), 22:5n-3 (**C**) and 22:6n-3 (**D**). * Significantly different from rats fed Control Diet. *p* < 0.05 were considered statistically significant. Groups are compared using Independent Samples t-test assuming equal variances.

Rats fed Baked Cod Diet had significantly higher level of 20:5n-3 in serum, liver, skeletal muscle and epididymal white adipose tissue when compared to rats fed the Control Diet (Figure 2). In addition, significantly higher levels of 22:5n-3 and 22:6n-3 were observed in serum and white adipose tissue from rats fed Baked Cod Diet when compared to controls. The level of 18:3n-3 was not affected in serum, liver, skeletal muscle and epididymal white adipose tissue.

Serum and white adipose tissue level of 20:4n-6 was significantly lower in Baked Cod Group, whereas the level of 22:5n-6 was significantly lower in serum, liver, muscle and white adipose tissue in rats in the Baked Cod Group when compared to the Control Group (Supplemental Table S1). Muscle and white adipose tissue levels of 22:4n-6 and muscle level of 18:2n-6 were significantly lower, whereas serum 20:3n-6 level was significantly higher in rats fed the Baked Cod Diet when compared to the control rats. The ratio of n-3 to n-6 PUFAs was significantly higher in serum, liver, skeletal muscle and epididymal white adipose tissue of rats fed Baked Cod Diet.

Also, saturated and monounsaturated fatty acids were affected by the Baked Cod Diet, with higher level of 14:0 in serum and liver, higher 16:0 in serum and muscle, higher 16:1n-7 in serum, higher 18:1n-9 in serum, and lower 18:1n-7 in liver, muscle and white adipose tissue.

Liver mRNA concentrations of FADS1 and FADS2 were significantly lower in Baked Cod Group when compared to Control Group, whereas SCD1 mRNA level was similar in these groups (Figure 1). Insulin is known to stimulate activities of FADS1 and FADS2, however no difference was seen between the groups for serum insulin concentration (*p* = 0.56, data not presented).

## 4. Discussion

Here we show for the first time that consumption of a diet containing 25% of protein from baked cod is sufficient to result in a lower serum cholesterol concentration and higher serum and tissue concentrations of long chain *n*-3 PUFAs in obese Zucker fa/fa rats, which is a hypercholesterolemic rat model of human obesity. Energy intake, growth, and relative weights of liver, thigh muscle and white adipose tissue were similar between rats fed the Baked Cod Diet or the Control Diet, which is of interest as an improvement of lipid concentrations after lean fish intake have mainly been explained by others as a result of weight loss when fish was included in an energy restricted diet [15,47]. To investigate possible mechanisms behind the lower circulating concentrations of total, HDL and LDL cholesterols, we measured hepatic mRNA concentrations of enzymes involved in cholesterol and bile acid synthesis. We also measured hepatic mRNA concentrations of fatty acid desaturases to see if biosynthesis of long chain PUFAs were affected by baked cod fillet intake. Our findings suggest that endogenous cholesterol synthesis as well as delta-5 and delta-6 desaturation of fatty acids were lower in rats fed Baked Cod Diet.

Circulating cholesterol is a strong predictor of vascular mortality, especially for ischemic heart disease [4], and lowering serum cholesterol concentration is an important strategy for reducing the risk of cardiovascular disease. Cholesterol concentrations in circulation, liver and extrahepatic tissues are controlled through the activities of HMG-CoA reductase (catalyses the rate-determining step in the biosynthetic pathway for cholesterol) and LDL receptor (facilitates uptake of LDL particles), and through faecal excretion of cholesterol and bile acids. In the present study, serum concentrations of total, HDL and LDL cholesterols in rats fed Baked Cod Diet were lower when compared to rats fed the Control Diet, despite a 3.5 times higher cholesterol content in the former diet. Studies in rats show that proteins from lean fish such as cod [27,28,29] and Alaska pollack [48,49,50,51,52], and also fat-free salmon protein hydrolysates [53,54], resulted in a lower concentration of circulating cholesterol when amounting to 100% or 50% of dietary protein intake. In contrast, circulating cholesterol concentrations were unaffected when Zucker fa/fa rats were fed 25% cod protein when compared to casein [41]. These contradictory reports underscore that different fish proteins may have different effects on cholesterol metabolism, and that more knowledge is warranted about how fish and fish proteins may affect cholesterol regulation in people with increased risk of developing hypercholesterolemia and for those who have already established hypercholesterolemia.

Lower circulating cholesterol has been associated with the amino acid compositions of dietary proteins, where lower ratios of methionine/glycine and lysine/arginine have shown a hypocholesterolemic effect in rats [53,55,56]. However, reports are conflicting regarding the effect of these ratios, as some report lower cholesterol in rats fed fish protein despite negligible differences in lysine/arginine ratio when compared to the control diets [52,57]. Since no differences were seen between the Baked Cod Diet and the Control Diet for these amino acid ratios, the hypocholesterolemic effect of the cod containing diet was most likely influenced by other factors. Faecal excretion of bile acids is the major route of cholesterol removal from the body, and taurine has been reported to have cholesterol lowering properties in rats by increasing the conversion of cholesterol to bile acids for faecal excretion [58,59]. Cholic acid can be conjugated with both glycine and taurine, but rats prefer taurine [60]. Since taurine was found only in the Baked Cod Diet this might be a mechanism by which dietary taurine could have affected the circulating cholesterol concentration through increasing the conjugation and thus faecal excretion of bile acids. Several studies have shown that rats fed 50 or 100% of protein from saithe or Alaska Pollack had lower serum cholesterol when compared to rats fed casein, which may be due to higher faecal bile acid excretion [49,50,52,61], however others report no changes in faecal excretion of cholesterol and bile acid after feeding 100% hydrolysed salmon protein [53,62]. In the present study, no differences were seen between the groups for serum and liver concentration of total bile acids, faecal excretion of cholesterol and bile acids, and mRNA concentration of CYP7A1, thus suggesting that another mechanism of action must be responsible for the lower serum concentrations of total, HDL and LDL cholesterols in rats fed Baked Cod Diet. 

The cholesterol content in the liver was similar between rats fed the Baked Cod Diet and the Control Diet, which is in contrast to our previous finding that obese Zucker rats fed cod proteins had higher hepatic cholesterol content when compared to rats fed a casein-based diet [41]. The hepatic HMGCR mRNA concentration was lower in Baked Cod Group, thus indicating that the endogenous production of cholesterol may have been lower when compared to Control Group. The finding of lower hepatic mRNA concentration of SOAT-2 in Baked Cod Group, which catalyses the esterification of cholesterol to cholesteryl ester, supports the assumption of a lower cholesterol synthesis in this group, and this is likely the mechanism behind the lower serum cholesterol concentrations in rats fed Baked Cod Diet. The lower LDLR mRNA concentration in liver from rats in Baked Cod Group may be a result of lower serum cholesterol concentration.

Cod fillet contains low amounts of fatty acids including long chain *n*-3 PUFAs, and thus the fatty acid composition in the Baked Cod Diet was mostly similar to that of the Control Diet. The long chain *n*-3 PUFAs 20:5n-3, 22:5n-3 and 22:6n-3 were found only in the Baked Cod Diet, albeit in very low amounts, but still the amounts of 20:5n-3, 22:5n-3 and 22:6n-3 in serum and tissues were different between the two experimental groups. Of particular interest is the higher level of 20:5n-3 in serum, liver, skeletal muscle and epididymal white adipose from rats fed the Baked Cod Diet when compared to those fed the Control Diet, as 20:5n-3 is a precursor for anti-inflammatory eicosanoids. In addition, rats fed the Baked Cod diet had higher levels of 22:5n-3 and 22:6n-3 and lower level of 20:4n-6 in serum and white adipose tissue when compared to controls, and the ratio of n-3 to n-6 PUFAs was higher in serum, liver, skeletal muscle and epididymal white adipose tissue. The higher level of long chain *n*-3 PUFAs in rats fed the Baked Cod Diet may reflect the *n*-3 PUFA content in this diet, as others have shown that a diet with very low content of salmon oil (0.75%) is sufficient to result in a higher amount of 22:6n-3 in liver microsomes and serum in obese Zucker rats [63]. This is in line with our recent paper showing that when obese Zucker rats were fed diets containing cod protein (25% of dietary protein) with a very low content of *n*-3 PUFAs (<0.1 wt %), the *n*-3/*n*-6 PUFA ratio in serum, liver and epididymal white adipose tissue was higher when compared to rats fed a casein based diet [41]. However, we have previously found that obese Zucker rats fed high dose fat-free salmon protein hydrolysate (100% of dietary protein) had higher amounts of 20:5n-3, 22:5n-3 and 22:6n-3 in liver, and more 20:5n-3 and 22:5n-3 in serum and phospholipids from triacylglycerol-rich lipoproteins [53], suggesting that the salmon protein hydrolysate may stimulate desaturation and elongation of 18:3n-3 to long chain *n*-3 PUFAs in these rats. Long-chain *n*-3 PUFAs are mainly produced from 18:3n-3 in the liver of rats, and the activities of the delta-6 and delta-5 desaturases are stimulated by insulin [64]. However, since no differences were seen between the groups for fasting serum insulin concentration and the mRNA concentrations of FADS1 and FADS2 were lower in liver from rats fed the Baked Cod Diet, it is most likely that the higher *n*-3 PUFA levels in serum, liver, skeletal muscle and epididymal white adipose tissue are caused by the marginally higher *n*-3 PUFA content in the Baked Cod Diet and are not a result of endogenous production.

The present study has some limitations. The obese Zucker fa/fa rat is a good model of human obesity and health related aspects of obesity [30], but still there are substantial differences between rats and humans that limit the reliability and translation of the present findings to humans. Humans transport the majority of cholesterol in LDL particles [65], while rats use HDL as the major cholesterol transporter [66]. Therefore, it is important to recognize that the observed effects in the current study could be specific for the obese Zucker fa/fa rat and not as apparent in humans.

The estimated average protein intake of Norwegian and US adults is 91–96 g/day [67,68], and if the recommendations to consume fish 2–3 times per week [69,70] are followed this will correspond to a weekly protein intake from fish of 60–90 g, or 9–13% of total protein intake. Most published studies on intake of fish proteins in rats have investigated the effects of a dietary intake of 100% fish protein [27,28,29,48,49,50,51,53,62], or a combination of 50% fish protein and 50% casein in the diet [52,54,57,61] compared to casein. It is not common, nor advised to consume protein from only one source in a healthy diet. A dietary intake of 25% fish protein of total protein intake would better reflect a normal consumption of protein from fish, and this relatively low dosage of protein from cod fillet was chosen to achieve a more realistic and safe intake of fish that is more relevant for intake in humans than using fish as the sole protein source. In rat studies, fish protein is often administered as hydrolysates whereas in a human diet, fish protein is commonly consumed as part of the whole fish fillet. Thus, the results from the current study are more relevant in relation to clinical trials compared to studies using 50 or 100% fish protein. 

## 5. Conclusions

The hypothesis of this study was that intake of baked cod fillet would lower serum cholesterol and increase contents of long chain *n*-3 PUFAs in serum and tissues in obese Zucker fa/fa rats. In line with our hypothesis, we found that when these rats consumed a diet containing 25% of protein from baked cod fillet this resulted in lower serum concentrations of total cholesterol, HDL cholesterol and LDL cholesterol. The beneficial effect on circulating cholesterol was probably mediated through down-regulation of endogenous cholesterol synthesis in liver, without affecting serum bile acid concentration and faecal excretion of cholesterol and bile acid. Also, Zucker fa/fa rats fed Baked Cod Diet had significantly higher levels of *n*-3 PUFAs in serum, liver, skeletal muscle and epididymal white adipose tissue when compared to rats fed the Control Diet, which is most likely explained by higher uptake of long-chain *n*-3 PUFAs from the diet and not a result of elevated desaturation of 18:3n-3. To conclude, obese Zucker fa/fa rats fed a diet containing baked cod fillet had lower serum cholesterol concentrations and higher contents of long chain *n*-3 PUFAs in serum, liver, skeletal muscle and epididymal WAT compared to rats fed casein as sole protein source.

## Figures and Tables

**Table 1:** Composition of the experimental diets.

g/kg Diet	Control Diet	Baked Cod Diet
Casein protein ^1^	216.22	162.16
Freeze dried baked cod ^2^	-	62.50
Corn starch	511.67	503.22
Sucrose	90.00	90.00
Cellulose	50.00	50.00
Soybean Oil	70.00	70.00
t-Butylhydroquinone	0.014	0.014
Mineral Mix (AIN-93-MX)	35.00	35.00
Vitamin Mix (AIN-93-VX)	10.00	10.00
L-Methionine	1.60	1.60
L-Cystine	3.00	3.00
Choline Bitartrate ^3^	2.50	2.50
Growth and Maintenance Supplement ^4^	10.00	10.00

^1^ Contains 92.5% crude protein, ^2^ Contains 80% crude protein, ^3^ Contains 41% choline, ^4^ Contains vitamin B12 (40 mg/kg) and vitamin K1 (25 mg/kg) mixed with sucrose (995 g/kg) and dextrose (5 g/kg).

**Table 2:** Contents of indispensable amino acids, the functional amino acid glycine, the conditionally essential amino acid arginine, taurine, the ratios of lysine/arginine and methionine/glycine, essential fatty acids and long-chain polyunsaturated fatty acids in the diets ^1.^

Per kg Diet	Control Diet	Baked Cod Diet
Amino Acids, g		
Arginine	6.4	7.1
Glycine	3.6	4.2
Histidine	5.1	4.8
Isoleucine	9.2	8.9
Leucine	16.0	16.0
Lysine	14.0	14.0
Methionine	6.4	6.8
Phenylalanine	9.1	8.8
Threonine	7.2	7.3
Valine	12.0	12.0
Taurine	ND	0.3
Lysine/ arginine ratio	2.2	2.0
Methionine/ glycine ratio	1.8	1.6
Fatty acids, g		
18:2n-6	32.7	30.4
18:3n-3	3.9	3.6
20:5n-3	ND	0.33
22:5n-3	ND	0.03
22:6n-3	ND	0.81
Cholesterol, mg	340	97

^1^ Means of two measurements, ND; not detected.

**Table 3:** Bodyweight at endpoint, body weight-to-square body length, relative organ weight, faecal output and energy intake.

	Control Group	Baked Cod Group	*p*
Bodyweight at endpoint, g	548 ± 32	566 ± 20	0.17
Body weight-to-square body length without tail ratio, kg/m^2^	9.8 ± 0.6	10.3 ± 0.4	0.062
Relative liver weight, g/100g body weight	4.5 ± 0.9	4.0 ± 0.6	0.11
Relative thigh muscle weight, g/100 g body weight	0.30 ± 0.04	0.29 ± 0.03	0.58
Relative amount of WAT ^1^, g/100 g body weight	7.4 ± 0.6	7.4 ± 0.5	0.97
Faecal output, g dry weight/24 h	2.8 ± 0.9	2.9 ± 0.9	0.76
Energy intake, kcal/kg bodyweight/24 h	266 ± 33	269 ± 51	0.91

Values are mean and standard deviations, n = 9 in Control group, and n = 10 in Baked Cod Group. *p* < 0.05 were considered significant. Groups are compared using Independent Samples t-test assuming equal variances. ^1^ The sum of epididymal, renal and retroperitoneal white adipose tissue from depots on right and left sides of the rat. WAT; white adipose tissue.

**Table 4:** Serum concentrations of cholesterols and total bile acids.

	Control Group	Baked Cod Group	*p*
Total cholesterol, mmol/L	6.6 ± 1.1	5.6 ± 0.9	0.032
HDL cholesterol, mmol/L	5.3 ± 0.6	3.8 ± 1.6	0.018
LDL cholesterol, mmol/L	1.7 ± 0.6	1.1 ± 0.5	0.0025
Total bile acids, umol/L	43 ± 24	29 ± 18	0.17

Values are mean and standard deviations, n = 9 in Control group, and n = 10 in Baked Cod Group. *p* < 0.05 were considered significant. Groups are compared using Independent Samples t-test assuming equal variances.

**Table 5:** Cholesterol and total bile acids in liver and faces, and cholesterol in skeletal muscle.

	Control Group	Baked Cod Group	*p*
Liver cholesterol, umol/g liver	9.3 ± 2.4	8.2 ± 1.9	0.27
Liver total bile acids, umol/g protein	15.6 ± 2.4	16.6 ± 2.4	0.38
Skeletal muscle cholesterol, umol/g	2.5 ± 1.1	2.3 ± 1.2	0.71
Faeces cholesterol, umol/24 h	21.2 ± 12.9	18.2 ± 5.3	0.52
Faeces total bile acids, umol/24 h	8.8 ± 2.7	10.4 ± 4.1	0.36

Values are mean and standard deviations, n = 9 in Control group, and n = 10 in Baked Cod Group. *p* < 0.05 were considered significant. Groups are compared using Independent Samples t-test assuming equal variances.

## Supplementary Materials

The following are available online at http://www.mdpi.com/2072-6643/10/7/840/s1, Table S1: Saturated, monounsaturated and n-6 polyunsaturated fatty acids, and ratio of n-3/n-6 polyunsaturated fatty acids in serum and tissues.
