# Supplementary material for: Intake of Baked Cod Fillet Resulted in Lower Serum Cholesterol and Higher Long Chain n-3 PUFA Concentrations in Serum and Tissues in Hypercholesterolemic Obese Zucker fa/fa Rats
Source: Nutrients. 2018 Jun 28;10(7):840. doi: 10.3390/nu10070840 (PMC6073601; doi:10.3390/nu10070840)
Supplement: Supplementary file 1 [file nutrients-10-00840-s001.pdf]

Supplemental Table 1: Saturated, monounsaturated and n-6 polyunsaturated fatty acids, and ratio of n-3/n-6 polyunsaturated fatty acids in serum and tissues

	Serum			Liver			Skeletal muscle			White adipose tissue		
	Control Group	Baked Cod Diet	P	Control Group	Baked Cod Diet	P	Control Group	Baked Cod Diet	P	Control Group	Baked Cod Diet	P
14:0	0.63 ± 0.10	0.84 ± 0.26	0.037	1.7 ± 0.2	1.9 ± 0.1	0.35	1.7 ± 0.4	1.8 ± 0.2	0.66	1.9 ± 0.1	2.0 ± 0.1	0.73
16:0	18.8 ± 0.9	21.3 ± 2.3	0.0073	36.6 ± 2.4	37.9 ± 1.8	0.19	27.6 ± 1.5	29.7 ± 1.3	0.0038	31.4 ± 1.0	31.7 ± 1.0	0.42
16:1 n-9	0.38 ± 0.05	0.42 ± 0.07	0.19	0.75 ± 0.12	0.68 ± 0.09	0.16	0.31 ± 0.04	0.28 ± 0.04	0.19	0.41 ± 0.03	0.39 ± 0.05	0.26
16:1 n-7	4.2 ± 0.7	5.2 ± 1.0	0.012	10.3 ± 0.9	10.5 ± 0.6	0.57	9.9 ± 3.0	10.3 ± 1.5	0.73	9.0 ± 1.3	9.0 ± 1.1	0.95
18:0	12.2 ± 0.9	11.6 ± 1.4	0.29	4.5 ± 1.0	4.6 ± 0.6	0.80	7.0 ± 2.4	6.2 ± 1.2	0.34	3.9 ± 0.5	3.9 ± 0.6	0.83
18:1 n-9	10.8 ± 1.2	13.8 ± 2.6	0.0070	27.7 ± 2.9	26.4 ± 1.7	0.26	20.5 ± 3.9	23.2 ± 2.2	0.073	30.8 ± 0.9	30.6 ± 1.0	0.58
18:1 n-7	2.8 ± 0.32	2.7 ± 0.2	0.45	4.6 ± 0.4	3.7 ± 0.6	0.0038	3.0 ± 0.2	2.8 ± 0.2	0.0012	2.9 ± 0.1	2.8 ± 0.1	0.037
18:2 n-6	11.6 ± 1.2	12.0 ± 2.5	0.64	7.3 ± 1.0	7.5 ± 1.8	0.72	15.9 ± 1.6	14.2 ± 1.5	0.025	15.9 ± 0.8	15.9 ± 1.4	0.89
18:3 n-6	0.34 ± 0.07	0.41 ± 0.09	0.069	0.20 ± 0.06	0.19 ± 0.04	0.70	0.067 ± 0.011	0.067 ± 0.008	0.87	0.074 ± 0.010	0.078 ± 0.009	0.49
20:3 n-6	0.85 ± 0.18	1.2 ± 0.3	0.0021	0.21 ± 0.10	0.31 ± 0.16	0.11	0.44 ± 0.16	0.45 ± 0.10	0.85	0.17 ± 0.02	0.18 ± 0.03	0.32
20:4 n-6	30.3 ± 2.1	22.3 ± 6.6	0.0027	3.3 ± 1.3	2.9 ± 0.7	0.34	5.5 ± 2.5	3.8 ± 1.3	0.072	0.44 ± 0.05	0.36 ± 0.03	$6.9x10^{-4}$
22:4 n-6	0.47 ± 0.08	0.45 ± 0.14	0.69	0.12 ± 0.04	0.11 ± 0.03	0.47	0.55 ± 0.19	0.38 ± 0.10	0.019	0.14 ± 0.01	0.12 ± 0.01	$1.5x10^{-4}$
22:5 n-6	0.45 ± 0.11	0.29 ± 0.08	0.0015	0.13 ± 0.05	0.08 ± 0.03	0.019	0.56 ± 0.26	0.31 ± 0.10	0.013	0.055 ± 0.007	0.042 ± 0.005	$2.6x10^{-4}$
n-3/n-6 ratio[1]	0.093 ± 0.011	0.156 ± 0.036	$9.6x10^{-5}$	0.14 ± 0.02	0.19 ± 0.02	$7.7x10^{-8}$	0.24 ± 0.05	0.28 ± 0.03	0.0036	0.107 ± 0.004	0.119 ± 0.006	$4.7x10^{-9}$

[1]the following fatty acids are included in the calculation; 18:3n-3, 20:5n-3, 22:5n-3, 22:6n-3, 18:2n-6, 18:3n-6, 20:3n-6, 20:4n-6, 22:4n-6, 22:5n-6
